# Systemic immune-inflammation index and in-stent restenosis in patients with acute coronary syndrome: a single-center retrospective study

**DOI:** 10.1186/s40001-024-01736-4

**Published:** 2024-02-26

**Authors:** Feng Xie, Zuozhong Yu, Yurong Xiong, Zhijian Wu, Yanqing Wu

**Affiliations:** Department of Cardiology, the Second Affiliated Hospital, Jiangxi Medical College, Nanchang University, No.1 Minde Road, Nanchang, Jiangxi 330006 People’s Republic of China

**Keywords:** In-stent restenosis, Drug-eluting stents, Systemic immune-inflammation index, Acute coronary syndrome, Percutaneous coronary intervention

## Abstract

**Background:**

In-stent restenosis (ISR) has been shown to be correlated with inflammation. This study aimed to examine the relationship between systemic immune-inflammation index (SII, an innovative inflammatory biomarker) and ISR in acute coronary syndrome (ACS) patients after drug-eluting stent (DES) implantation.

**Methods:**

Subjects who were diagnosed with ACS and underwent DES implantation were enrolled retrospectively. All individuals underwent follow-up coronary angiography at six to forty-eight months after percutaneous coronary intervention (PCI). SII was defined as [(platelet count × neutrophil count)/lymphocyte count], and Ln-transformed SII (LnSII) was carried out for our analysis. Multivariate logistic regression analysis was employed to assess the association between LnSII and DES-ISR.

**Results:**

During a median follow-up period of 12 (11, 20) months, 523 ACS patients who underwent follow-up angiography were included. The incidence of DES-ISR was 11.28%, and patients in the higher LnSII tertile trended to show higher likelihoods of ISR (5.7% vs. 12.1% vs. 16.0%; *P* = 0.009). Moreover, each unit of increased LnSII was correlated with a 69% increased risk of DES-ISR (OR = 1.69, 95% CI 1.04–2.75). After final adjusting for confounders, a significant higher risk of DES-ISR (OR = 2.52, 95% CI 1.23–5.17) was found in participants in tertile 3 (≥ 6.7), compared with those in tertiles 1–2 (< 6.7). Subgroup analysis showed no significant dependence on age, gender, body mass index, current smoking, hypertension, and diabetes for this positive association (all *P* for interaction > 0.05).

**Conclusion:**

High levels of SII were independently associated with an increased risk of DES-ISR in ACS patients who underwent PCI. Further prospective cohort studies are still needed to validate our findings.

**Supplementary Information:**

The online version contains supplementary material available at 10.1186/s40001-024-01736-4.

## Background

Percutaneous coronary intervention (PCI) with drug-eluting stent (DES) implantation is the main revascularization strategy for patients with acute coronary syndrome (ACS). However, despite continued improvement in stent technology and anti-platelet therapy, in-stent restenosis (ISR) remains an important issue that limits the clinical safety and efficacy of DES. Previous studies have shown that the cumulative incidence of DES-ISR in real-world practice reaches up to 10% at 5 years following PCI [1, 2]. ISR may lead to the recurrence of adverse cardiovascular outcomes, such as myocardial infarction and sudden cardiac death. Hence, early identification of accurate and convenient biomarkers of DES-ISR has important clinical significance for ACS patients.

Inflammation has been well studied as a potential cardiovascular risk factor [3]. Accumulated evidence suggests that inflammatory reactions are correlated with atherosclerotic cardiovascular diseases, such as arterial hypertension, ischemic stroke, and coronary artery disease (CAD) [4–6]. Notably, a growing number of studies also show that inflammation plays a pivotal role in the genesis and advancement of ISR [7, 8]. Inflammatory factors could induce endothelial regeneration and proliferation, leading to restenosis [9, 10]. Indeed, many studies have demonstrated that inflammatory indices such as high-sensitivity C-reactive protein, neutrophil/lymphocyte ratio (NLR), and eosinophil cationic protein are associated with a high risk of ISR [11, 12]. As a result, the correlation between inflammation and ISR has garnered escalating scholarly interest.

The systemic immune-inflammation index (SII) is an innovative and integrated inflammatory biomarker based on three types of immune cells (platelets, neutrophils, and lymphocytes). Many studies have confirmed its high prognostic values in chronic heart failure, ischemic stroke, and CAD, which suggests potential implications for cardiovascular disease (CVD) [13–15]. Moreover, SII had better performance than platelet/ lymphocyte ratio (PLR) and NLR in predicting the poor outcomes of ACS and the hemodynamically severe obstruction of chronic coronary syndrome (CCS) [16, 17]. Thus, SII may be a superior and comprehensive indicator of local immune responses and systemic inflammation [18]. Studies have shown that blood cell parameters (such as PLR, NLR, and platelet distribution width) are correlated with an increased risk of ISR in patients with angina pectoris and coronary chronic total occlusion lesions [12]. However, there are no studies exploring the relationship between SII and DES-ISR, especially in ACS patients.

Taken together, it is of great significance to examine the relationship between SII, an economical and superior indicator, and ISR for evaluating intravascular conditions and improving the prognosis of CAD more effectively. To address this knowledge gap, we therefore designed this research to investigate the association between SII and ISR in ACS patients after DES-based PCI.

## Methods

### Study population

From January 2019 to October 2020, subjects who were diagnosed with ACS and underwent DES implantation in the Second Affiliated Hospital of Nanchang University were enrolled retrospectively. Inclusion criteria were: (1) age over 18 years old; (2) diagnosed as ACS; and (3) received follow-up coronary angiography (CAG) between six and forty-eight months after PCI. The indication for follow-up CAG is a comprehensive assessment by the clinician based on the patient's condition, typically one year after the procedure. Meanwhile, subjects meeting any of the following criteria were eliminated: (1) severe hepatic and renal dysfunction; (2) acute/chronic inflammatory disease; (3) treatment of the culprit lesion with a bare metal stent or balloon angioplasty; and (4) combined with malignant tumors or a life expectancy of < 6 months.

Totally, 545 patients with ACS who satisfied the inclusion and exclusion criteria were enrolled at first; after excluding individuals with missing data on SII (n = 22), 523 eligible patients were included in our final analysis. This study was performed in line with the Declaration of Helsinki, and Ethic Committee approval was obtained from the Second Affiliated Hospital of Nanchang University. All participants provided their written informed consent upon admission.

### Data collection

Demographic and clinical features, including gender, age, smoking status, body mass index (BMI), past medical history, angiographic findings, laboratory tests, and medication at discharge (secondary prevention strategies), were obtained from the medical system by trained personnel. Peripheral blood samples were collected (before coronary angiography) after overnight fasting (> 8 h) for laboratory examinations. Then, serum uric acid (SUA), fasting blood glucose (FBG), hemoglobin A1c (HbA1c), homocysteine (Hcy), total cholesterol (TC), triglycerides (TG), low-density lipoprotein-C (LDL-C), and high-density lipoprotein-C (HDL-C) were measured with standard assays. The SUA was determined by a direct enzyme method (Medical Co., Ltd., Ningbo, China), the FBG was determined with the hexokinase/glucose-6-phosphate dehydrogenase method (Biote Co., Ltd., Nanchang, China), and the Hcy was determined by enzymatic methods using test kits (AUSA Co., Ltd., Shenzhen, China). The TC was measured using the enzymatic colorimetric method (Medical Co., Ltd., Ningbo, China), the TG was measured using the GPO-POD method (Beckman Coulter, Suzhou, China), and the LDL-C and HDL-C were measured by direct homogeneous assay methods using detergents (Medical Co., Ltd., Ningbo, China). All biochemical parameters were measured in the central laboratory of the Second Affiliated Hospital of Nanchang University using an automated analyzer (Olympus AU 2700).

The estimated glomerular filtration rate (eGFR) was determined using the Chronic Kidney Disease Epidemiology Collaboration creatinine equation: eGFR = 142 × min (serum creatinine/κ, 1)^α^ × max (serum creatinine/κ, 1)^−1.200^ × 0.9938^age^ × 1.012 (if female) (κ: female = 0.7, male = 0.9; α: female = -0.241, male = -0.302) [19]. BMI was calculated as weight (kg) divided by height squared (m^2^), and current smoking was defined as the daily use of one or more cigarettes for at least 1 year. Hypertension was defined as a blood pressure (BP) ≥ 140/90 mmHg or the use of anti- hypertensive drugs. Hyperlipidemia was defined as a fasting TG ≥ 2.26 mmol/L, TC > 6.22 mmol/L, LDL-C > 4.14 mmol/L, or receiving lipid-lowering drugs. Diabetes was defined as a FBG > 7.0 mmol/L, random blood glucose > 11.1 mmol/L, or the use of glucose-lowering medication. Besides, chronic kidney disease was confirmed based on the presence of kidney damage or an eGFR < 60 ml/min per 1.73 m^2^ for at least 3 months.

### Exposure variable and outcomes

The complete blood counts were measured by trained medical personnel using automated hematology analyzing devices. Lymphocyte, platelet, and neutrophil counts were employed for our analysis. Accordingly, SII (× 10^3^ cells/μl) as an exposure variable was calculated as [(platelet count × neutrophil count)/lymphocyte count] [18].

The outcome measure of this study was the incidence of ISR. It was defined as stenosis of a segment inside the stent or its 5-mm edges with a diameter stenosis of more than 50% [20, 21]. All individuals underwent follow-up CAG between six and forty-eight months after the successful baseline PCI. Then, two independent and experienced cardiologists who were not aware of the study's purpose interpreted the CAG results. Besides, all subjects received current guidelines-recommended secondary prevention for CAD.

### Statistical analysis

All statistical analyses were performed using Empower software (www.empowerstats.com; X&Y Solutions, Inc., Boston, MA) and R version 3.4.3 (http://www.R-project.org, The R Foundation). Based on the data distribution, continuous variables are described as means (standard deviations) or medians (interquartile ranges). Comparisons among groups were conducted by one-way analysis of variance or the Kruskal-Wallis test. Categorical variables are described as numbers (percentages), and comparisons among groups were conducted by the chi-squared test or Fisher’s exact 2 × 2 test.

Because SII had a strongly skewed distribution, the Ln-transformed SII (LnSII) was used for our analysis. Multivariate logistic regression analysis was employed to assess the relationship between LnSII and DES-ISR in different models. Model 1 was not adjusted for covariates; model 2 was adjusted for age, gender, and BMI; and model 3 was adjusted for age, gender, BMI, TC, LDL-C, hypertension, diabetes, length of stents, and minimal stent diameter. The covariates were selected based on the matched odds ratio (OR) changed at least 10% when added to this model [22]. We also converted LnSII into tertiles for sensitivity analyses. Furthermore, a generalized additive model and a fitted smoothing curve were used to explore the shape of the curve or the linear relationship between LnSII and DES-ISR. Subgroup analysis stratified by age (< 65/ ≥ 65 years), gender (male/female), BMI (< 25/ ≥ 25 kg/m^2^), current smoking (yes/no), hypertension (yes/no) and diabetes (yes/no) was also performed. Besides, a receiver operating characteristic (ROC) curve was used to assess the ability of PLR, NLR, and SII to identify DES-ISR in patients with ACS. Additionally, we used multiple imputation (MI), based on 5 replications and the Markov-chain Monte Carlo method in the SAS MI procedure, to account for missing data on TC, LDL-C, Hcy, HbA1c, and smoking status. Besides, the sample size was determined based on the power analysis. A two-tailed *P* value < 0.05 was considered significant.

## Results

### Baseline characteristics

The study included 523 patients with ACS undergoing follow-up CAGs over a median follow-up period of 12 (11, 20) months. The average age of the overall cohort was 65.70 ± 10.33 years, and 397 (75.91%) were male. According to the LnSII tertiles, subjects were divided into three subgroups. As illustrated in Table 1, patients in the highest LnSII subgroup were more likely to have higher FPG, TC, triglycerides, LDL-C, and homocysteine; to have a higher proportion of hypertension prevalence and the use of oral hypoglycemic drugs; and to have a lower level of serum albumin (all *P* < 0.05). Although the proportions of current smoking, hyperlipidemia, and diabetes were higher in the T_3_ subgroup than in the other two subgroups, the difference was not statistically significant.

**Table 1:** Baseline characteristics of patients stratified by tertiles of LnSII

Variables^#^	Tertile categories of LnSII			*P* value
	T_1_ (n = 174)	T_2_ (n = 174)	T_3_ (n = 175)	
Demographics				
Age (years)	66.25 ± 9.34	65.86 ± 9.97	64.99 ± 11.56	0.511
Male, n (%)	125 (71.84%)	132 (75.86%)	140 (80.00%)	0.204
BMI (kg/m^2^)	24.19 ± 3.03	24.14 ± 3.30	24.32 ± 2.65	0.881
Current smoking, n (%)	75 (44.12%)	73 (43.45%)	92 (53.18%)	0.131
Medical history				
Hypertension, n (%)	20 (11.49%)	33 (18.97%)	44 (25.14%)	0.005
Hyperlipidemia, n (%)	4 (2.30%)	8 (4.60%)	12 (6.86%)	0.126
Diabetes, n (%)	53 (30.46%)	39 (22.41%)	56 (32.00%)	0.103
Chronic kidney disease, n (%)	3 (1.72%)	7 (4.02%)	5 (2.86%)	0.438
Laboratory tests				
eGFR (ml/min/1.73m^2^)	80.28 ± 18.08	77.85 ± 23.56	78.97 ± 22.13	0.570
FPG (mmol/L)	5.97 ± 2.21	5.91 ± 2.12	7.09 ± 3.31	< 0.001
HbA1c (%)	6.19 ± 1.22	6.27 ± 1.25	6.40 ± 1.33	0.504
SUA (umol/L)	367.85 ± 95.11	377.40 ± 101.69	385.40 ± 121.05	0.308
Albumin, serum (g/L)	38.69 ± 3.47	37.85 ± 3.39	37.19 ± 3.55	< 0.001
TC (mmol/L)	4.01 ± 0.87	4.38 ± 1.02	4.69 ± 1.00	< 0.001
Triglycerides (mmol/L)	1.50 ± 0.74	1.67 ± 1.12	1.84 ± 1.11	0.010
HDL-C (mmol/L)	1.05 ± 0.29	1.03 ± 0.23	1.00 ± 0.24	0.250
LDL-C (mmol/L)	2.33 ± 0.69	2.67 ± 0.89	2.93 ± 0.83	< 0.001
Homocysteine (umol/L)	14.29 ± 5.36	15.27 ± 7.08	16.23 ± 8.07	0.040
Angiographic findings				
Chronic total occlusions, n (%)	23 (13.22%)	19 (10.92%)	26 (14.86%)	0.547
Number of stent (/patients)	2 (1, 2)	2 (1, 2)	1 (1, 2)	0.095
Length of stents (mm/patients)	26.88 ± 6.30	26.81 ± 6.36	27.64 ± 5.98	0.381
Minimal stent diameter (mm)	3.05 ± 0.44	3.01 ± 0.49	3.02 ± 0.43	0.713
Medications at discharge				
Aspirin, n (%)	174 (100.00%)	174 (100.00%)	175 (100.00%)	> 0.99
Clopidogrel/ticagrelor, n (%)	174 (100.00%)	174 (100.00%)	175 (100.00%)	> 0.99
Statin, n (%)	174 (100.00%)	172 (98.85%)	173 (98.86%)	0.366
β-block, n (%)	151 (86.78%)	163 (93.68%)	155 (88.57%)	0.090
ACEI/ARB, n (%)	115 (66.09%)	114 (65.52%)	130 (74.29%)	0.142
Oral hypoglycemic drugs, n (%)	42 (24.14%)	26 (14.94%)	50 (28.57%)	0.008

*SII* systemic immune-inflammation index, *BMI* body mass index, *eGFR* estimated glomerular filtration rate, *FPG* fasting plasma glucose, *HbA1c* hemoglobin A1c, *TC* total cholesterol, *HDL-C* high-density lipoprotein-C, *LDL-C* low-density lipoprotein-C, *SUA* serum uric acid, *ACEI/ARB* angiotensin-converting enzyme inhibitor/angiotensin receptor blocker

^#^Data are shown as mean ± SD, median (IQR), or n (%)

Additionally, the baseline characteristics of non-ISRs (N = 464) and ISRs (N = 59) were summarized in Additional file 1: Table S1. Compared with the non-ISR subgroup, subjects in the ISR subgroup had elevated concentrations of FPG, TC, and LDL-C, as well as statistically significant differences in eGFR and serum albumin (all *P* < 0.05). In regards to the procedure details, ISR patients had higher proportions of chronic total occlusions, a higher number of stents, and a smaller diameter of stent (all *P* < 0.05).

### Association of SII with in-stent restenosis

**Fig. 1:**
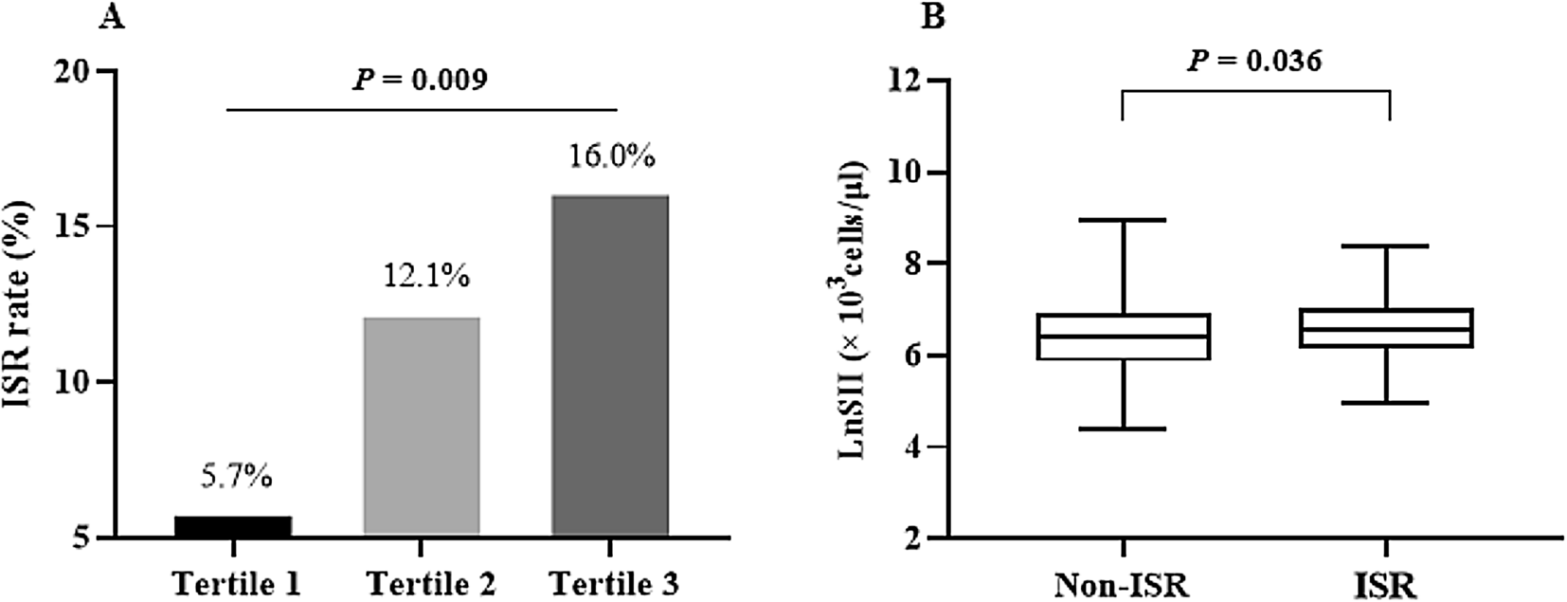
**A** The impacts of LnSII on the incidence of DES-ISR and **B** the comparison of LnSII between the non-ISR and ISR subgroups. Abbreviations: SII, systemic immune-inflammation index; DES, drug-eluting stent; ISR, in-stent restenosis

The incidence of DES-ISR was 11.28% (59/523) for the overall population, and subjects in the higher LnSII tertile trended to show higher likelihoods of DES-ISR (5.7% vs. 12.1% vs. 16.0%, *P* = 0.009; Fig. 1A). Additionally, LnSII was also significantly higher in the ISR subgroup than in the non-ISRs (6.66 ± 0.70 vs. 6.44 ± 0.75; Fig. 1B), with a statistical difference (*P* = 0.036).

The results of multivariable logistic regression for the impacts of LnSII on DES-ISR are shown in Table 2. A positive association between LnSII (per 1-unit increase) and DES-ISR was detected (Model 1: OR = 1.45, 95% CI 1.02–2.05, *P* = 0.037; Model 2: OR = 1.77, 95% CI 1.17–2.66, *P* = 0.006). Moreover, in the case of fully adjusted covariates, the results demonstrated that each unit of increased LnSII was correlated with a 69% increased risk of DES-ISR (Model 3: OR = 1.69, 95% CI 1.04–2.75, *P* = 0.034). When LnSII was analyzed as a categorical variable, the association persisted in different models (Table 2). After final adjusting for confounders in Model 3, a significant higher risk of DES-ISR (OR = 2.52, 95% CI 1.23–5.17, *P* = 0.011) was found in participants in tertile 3 (≥ 6.7), compared with those in tertiles 1–2 (< 6.7). Using imputation data, we reanalyzed the relationship between LnSII and DES-ISR and did not find any qualitative differences (Additional file 1: Table S2).

**Table 2:** Association of LnSII with DES-ISR in multivariable logistic regression models

LnSII	Events (%)	DES-ISR, OR (95% CI), *P* value		
		Model 1	Model 2	Model 3
Per 1-unit increase	59 (11.3%)	1.45 (1.02, 2.05), 0.037	1.77 (1.17, 2.66), 0.006	1.69 (1.04, 2.75), 0.034
Tertiles				
T_1_ (< 6.1)	10 (5.8%)	Ref.	Ref.	Ref.
T_2_ (6.1–6.7)	21 (12.1%)	2.25 (1.03, 4.93), 0.042	2.46 (0.98, 6.21), 0.054	1.87 (0.70, 4.98), 0.213
T_3_ (≥ 6.7)	28 (16.0%)	3.12 (1.46, 6.65), 0.003	4.50 (1.83, 11.08), 0.001	3.69 (1.40, 9.70), 0.008
* P* for trend		0.003	< 0.001	0.006
Categories				
T_1-2_ (< 6.7)	31 (8.9%)	Ref.	Ref.	Ref.
T_3_ (≥ 6.7)	28 (16.0%)	1.95 (1.13, 3.36), 0.016	2.63 (1.38, 5.01), 0.003	2.52 (1.23, 5.17), 0.011

Model 1: crude model

Model 2: adjusted for age, gender, and body mass index

Model 3: adjusted for age, gender, body mass index, total cholesterol, low-density lipoprotein-C, hypertension, diabetes, length of stents, and minimal stent diameter. The covariates were selected based on the matched odds ratio changed at least 10% when added to this model

*OR* odds ratio, *CI* confidence interval *DES* drug-eluting stent, *ISR* in-stent restenosis, *SII* systemic immune-inflammation index

**Fig. 2:**
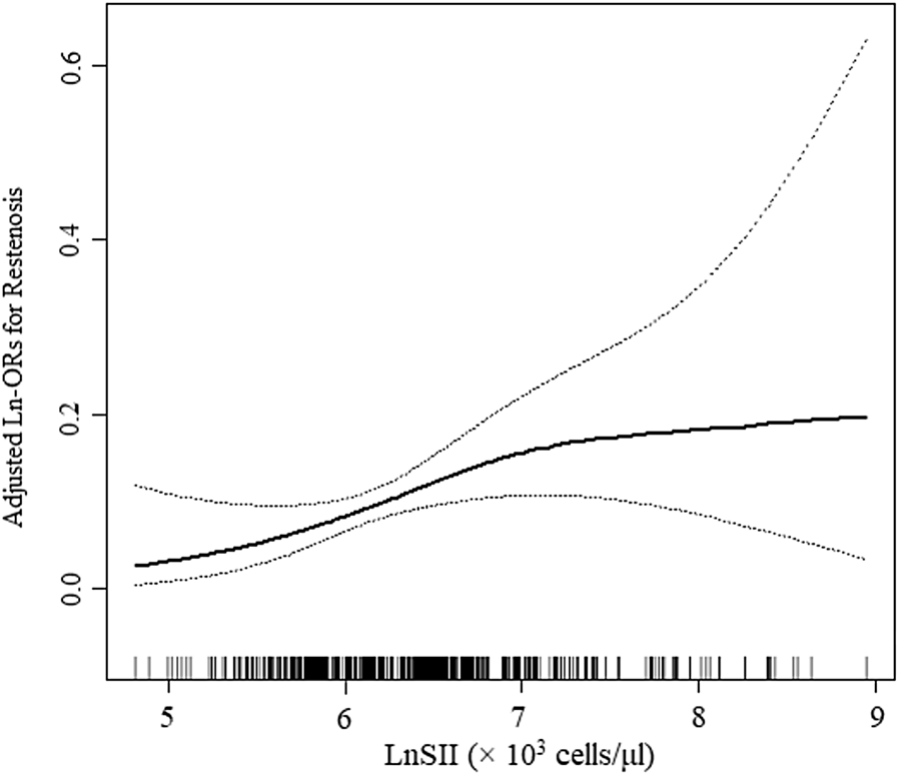
Smooth curve fitting for LnSII and DES-ISR. **A** linear relationship between LnSII and the risk of DES-ISR was detected by the generalized additive model. The solid line and dashed line represent the estimated values and their corresponding 95% confidence interval

Moreover, smooth curve fitting showed no non-linear relationship between LnSII and DES-ISR in the entire population (Fig. 2).

### Subgroup analysis

**Fig. 3:**
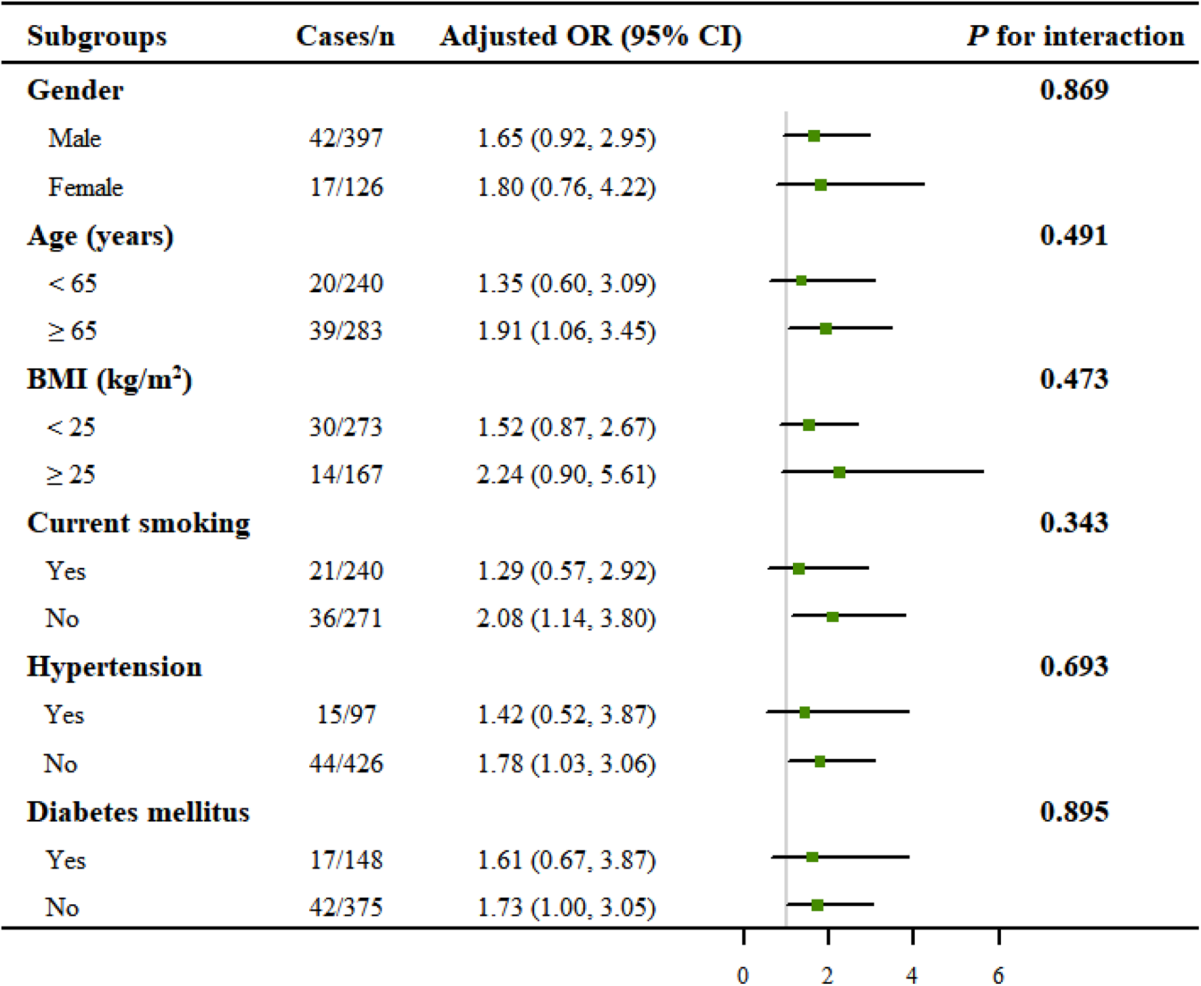
Subgroup analysis for the association between LnSII and DES-ISR. All presented covariates were adjusted (as Model 3) except the corresponding stratification variable. None of the stratifications, including gender, age, BMI, current smoking, hypertension, and diabetes, significantly affected the positive association between LnSII and DES-ISR

To evaluate the robustness of the relationship between LnSII and DES-ISR, subgroup analyses were further performed. As demonstrated in Fig. 3, none of the stratifications, including age, gender, BMI, current smoking, hypertension, and diabetes, significantly affected the positive association between LnSII and DES-ISR (all *P* for interaction > 0.05). These results indicated that the positive association was similar in different subgroups and could be appropriate for various population settings as well.

### ROC curves between SII and other systemic immune indices

The ROC curve was employed to assess the ability of NLR, PLR, and SII to identify DES-ISR in ACS patients. As presented in Additional file 1: Figure S1, the area under the curve (AUC) of SII for the identification of DES-ISR was 0.600 (95% CI 0.529–0.672), higher than that of NLR (0.589, 95% CI 0.511–0.668) and PLR (0.577, 95% CI 0.500–0.655). The optimal cut-off value of SII to detect DES-ISR was > 417.9 (× 10^3^ cells/μl), with 86.4% sensitivity and 34.5% specificity (*P* < 0.05). These findings indicate that SII is superior to other systemic immune indices.

## Discussion

To our knowledge, this is the first report to investigate the association between SII and ISR in patients with ACS undergoing DES-based PCI. The results demonstrated that SII was significantly associated with an increased risk of DES-ISR after adjusting for covariates including demographics, CVD risk factors, angiographic features, and others, presenting a nearly linear dose-response relationship. The highest LnSII subgroup (≥ 6.7 × 10^3^ cells/μl) was 2.52-fold correlated with DES-ISR as compared with the lowest LnSII subgroup (< 6.7 × 10^3^ cells/μl). Moreover, the ROC curve showed that SII had better identification performance for DES-ISR than other systemic immune indices (NLR and PLR). These findings indicated that SII may be a promising predictor for evaluating ISR after DES-PCI in ACS patients.

First proposed by Hu et al. in 2014, SII was initially used to assess the immune-inflammatory status and the risk of mortality in patients with malignancies [18, 23, 24]. However, accumulating evidence suggested that SII was also related to adverse clinical outcomes in diseases other than malignancies, particularly atherosclerotic cardiovascular disease [25–28]. For example, Candemir et al. examined the association between SII and CAG findings in 669 patients with stable angina pectoris. The results indicated that SII was significantly associated with the severity of CAG and high SYNTAX scores [26]. Another retrospective cohort study indicated that high SII levels could predict high 30- and 90-day mortalities as well as a high risk of major cardiovascular adverse events (MACEs) in patients with congestive heart failure [27]. Recently, Zheng et al. carried out a retrospective study of 887 myocardial infarction patients, which showed that SII ≥ 636 was an independent risk factor for intra-stent thrombosis after coronary stent implantation [28]. However, no relevant data regarding the effect of SII on ISR after DES-based PCI is known. The present study offered a timely opportunity to evaluate the dose-response relationship between SII and ISR, revealing that SII was also an independent risk factor for DES-ISR in patients with ACS. Our subjects included patients 48 months after PCI, suggesting that SII may have potential long-term predictive value. Based on our findings, timely interventions for patients with elevated SII levels may reduce the incidence of DES-ISR.

By integrating three circulating immune cells, consisting of neutrophils, platelets, and lymphocytes, SII can assess the systemic immune and inflammatory status. An increase in the level of SII indicates a high immune-inflammatory status in the human body, which may correlate with the development of multiple inflammation-related diseases [29–32]. A cross-sectional study of 22,290 individuals from the 1999–2010 National Health and Nutrition Examination Survey found that the OR for hypertension prevalence per In-transformed increment in SII was estimated at 1.115 (95% CI 1.045–1.188) [33]. Moreover, recent evidence also indicates that higher levels of SII are correlated with a higher risk of hyperlipidemia (OR = 1.02; 95% CI 1.00–1.04) and diabetes (OR = 2.024; 95% CI 1.297–3.157) [34, 35]. Consistent with most research, our current work revealed that elevated SII was correlated with increased levels of FPG, TC, triglycerides, LDL-C, and homocysteine, a decreased level of serum albumin, and a high prevalence of hypertension, which may have contributed to the development of ISR [36–39]. As expected, SII, either as a continuous or categorical variable, was independently related to an increased risk of DES-ISR. Thus, taking SII into consideration may have important clinical implications for optimizing the early risk stratification of ISR in ACS patients.

Since SII is derived from lymphocyte, neutrophil, and platelet counts, it may be considered a modified and powerful combination of NLR and PLR [23, 40]. Indeed, the ROC curve showed that SII had better identification performance for DES-ISR than NLR and PLR (AUC: 0.600 vs. 0.589 and 0.577). However, the exact mechanisms underlying the association of SII with DES-ISR are unknown, and the following explanations can be considered. The increase in SII suggests either a relative increase in neutrophil and platelet counts or a relative decrease in lymphocyte counts. Neutrophils could lead to oxidative stress and endothelial dysfunction by releasing large amounts of myeloperoxidase and nicotinamide adenine dinucleotide phosphate oxidase [41]. Meanwhile, platelets interacted with neutrophils and lymphocytes to induce monocyte adhesion and transport, release inflammatory factors (such as interleukin-6 and tumor necrosis factor alpha), and ultimately promote local inflammation [42]. The interaction of inflammation and platelet activation promotes the formation of neointima and atherosclerosis, which in turn leads to the development of ISR [42, 43]. Besides, our study and previous evidence indicate that SII is closely associated with multiple cardiometabolic risk factors (such as FPG, triglycerides, and serum albumin) [44, 45], which may also contribute to this relationship.

Some strengths of our study can be identified. This study explores for the first time the relationship between SII and DES-ISR in ACS patients and reveals a nearly linear dose-response relationship. Although the findings showed a relatively high rate of ISR (11.28%) at 1 year follow-up, this may be related to the higher proportion of acute ST-segment elevation myocardial infarction (47.3%, data not shown) and current smoking (45.9%) [46, 47]. Moreover, we adjusted for many potential confounders to produce more reliable findings. We also handled SII as both a continuous and categorical variable, which reduced the contingency in the data analysis and improved the robustness of the results. Besides, the subgroup analyses indicated that the positive SII-ISR association was similar in various population settings. Hence, SII can be used as an inexpensive and practical method to screen for DES-ISR risk in patients with ACS in clinical settings, especially in underdeveloped areas.

## Limitations

Despite that, there were certain limitations to our study. First, this is a single-center and retrospective study with a small sample size, which may cause some deviations in the results. Second, this study enrolled only patients with ACS, which affects the generalizability of the findings to CCS patients. Third, although SII had better identification performance for DES-ISR than other novel systemic indices (NLR and PLR), we did not evaluate its superiority compared to traditional inflammatory markers such as C-reactive protein. We also did not consider the immune responses of patients after recovering from COVID-19, even though all patients tested negative for COVID-19 at admission. Last, we assessed SII only once after admission and did not collect information on the changes in SII during follow-up.

## Conclusion

Our study indicated that SII was significantly and positively associated with the risk of DES-ISR in ACS patients after successful PCI, presenting a nearly linear dose–response relationship. Moreover, SII had better identification performance for DES-ISR than other systemic immune indices, including NLR and PLR. Despite that, further prospective cohort studies are still needed to validate our findings.

### Supplementary Information

**Additional file 1: Table S1.** Baseline characteristics of patients with and without ISR. **Table S2.** Multiple-imputation analysis which is based on 5 replications and the Markov-chain Monte Carlo method in the SAS multiple imputation procedure†. **Figure S1.** The ROC curve of SII, NLR and PLR for the identification of DES-ISR in patients with ACS. Abbreviations: ROC, receiver operator characteristic; SII, systemic immune inflammation index; NLR, neutrophil/lymphocyte ratio; PLR, platelet to lymphocyte ratio; DES, drug-eluting stent; ISR, in-stent restenosis; ACS, acute coronary syndrome.

## Data Availability

The data that support the findings of this study are available from the corresponding author upon reasonable request.

## Abbreviations

ACS: Acute coronary syndrome
PCI: Percutaneous coronary intervention
CAG: Coronary angiography
DES: Drug-eluting stent
ISR: In-stent restenosis
CAD: Coronary artery disease
CVD: Cardiovascular disease
NLR: Neutrophil/lymphocyte ratio
PLR: Platelet-to-lymphocyte ratio
SII: Systemic immune-inflammation index
BMI: Body mass index
eGFR: Estimated glomerular filtration rate
FBG: Fasting blood glucose
SUA: Serum uric acid
HbA1c: Hemoglobin A1c
TC: Total cholesterol
TG: Triglycerides
LDL-C: Low-density lipoprotein-C
HDL-C: High-density lipoprotein-C
Hcy: Homocysteine
OR: Odds ratio
ROC: Receiver operating characteristic
MI: Multiple imputation
AUC: Area under the curve
MACEs: Major cardiovascular adverse events
CCS: Chronic coronary syndrome
