# Supplementary material for: Systemic immune-inflammation index and in-stent restenosis in patients with acute coronary syndrome: a single-center retrospective study
Source: Eur J Med Res. 2024 Feb 26;29:145. doi: 10.1186/s40001-024-01736-4 (PMC10895800; doi:10.1186/s40001-024-01736-4)
Supplement: Supplementary file 1 — Additional file 1: Table S1. Baseline characteristics of patients with and without ISR. Table S2. Multiple-imputation analysis which is based on 5 replications and the Markov-chain Monte Carlo method in the SAS multiple imputation procedure†. Figure S1. The ROC curve of SII, NLR and PLR for the identification of DES-ISR in patients with ACS. Abbreviations: ROC, receiver operator characteristic; SII, systemic immune inflammation index; NLR, neutrophil/lymphocyte ratio; PLR, platelet to lymphocyte ratio; DES, drug-eluting stent; ISR, in-stent restenosis; ACS, acute coronary syndrome. [file 40001_2024_1736_MOESM1_ESM.doc]

**Systemic immune-inflammation index and in-stent restenosis in patients with acute coronary syndrome: A single-center retrospective study**

Feng Xie, MD, PhD ^1^, Zuozhong Yu, MD, PhD ^1^, Yurong Xiong, MD, PhD ^1^,

Zhijian Wu, MD, PhD ^1^, Yanqing Wu, MD, PhD ^1,*^

Table S1. Baseline characteristics of patients with and without ISR

| **Variables^#^** | **Non-ISR subgroup**  **(n=464)** | **ISR subgroup**  **(n=59)** | ***P* value** |
| --- | --- | --- | --- |
| **Demographics** |  |  |  |
| Age (years) | 65.42 ± 10.49 | 67.92 ± 8.67 | 0.080 |
| Male, n (%) | 109 (23.49%) | 17 (28.81%) | 0.368 |
| BMI (kg/m^2^) | 24.25 ± 3.06 | 23.85 ± 2.63 | 0.402 |
| Current smoking, n (%) | 219 (48.24%) | 21 (36.84%) | 0.104 |
| **Medical history** |  |  |  |
| Hypertension, n (%) | 82 (17.67%) | 15 (25.42%) | 0.149 |
| Hyperlipidemia, n (%) | 22 (4.74%) | 2 (3.39%) | 0.640 |
| Diabetes, n (%) | 131 (28.23%) | 17 (28.81%) | 0.926 |
| Chronic kidney disease, n (%) | 12 (2.59%) | 3 (5.08%) | 0.279 |
| **Laboratory tests** |  |  |  |
| eGFR (ml/min/1.73m^2^) | 79.74 ± 21.23 | 73.43 ± 21.80 | 0.032 |
| FPG (mmol/L) | 6.24 ± 2.51 | 7.02 ± 3.61 | 0.036 |
| HbA1c (%) | 6.24 ± 1.19 | 6.51 ± 1.72 | 0.128 |
| SUA (umol/L) | 376.06 ± 104.34 | 383.47 ± 123.52 | 0.616 |
| Albumin, serum (g/L) | 38.11 ± 3.35 | 36.47 ± 4.37 | <0.001 |
| TC [(mmol/L)](https://wwwn.cdc.gov/Nchs/Nhanes/2013-2014/BIOPRO_H.htm#LBDSCHSI) | 4.30 ± 0.95 | 4.78 ± 1.28 | <0.001 |
| [Triglycerides (mmol/L)](https://wwwn.cdc.gov/Nchs/Nhanes/2013-2014/BIOPRO_H.htm#LBDSTRSI) | 1.65 ± 0.98 | 1.86 ± 1.23 | 0.137 |
| HDL-C (mmol/L) | 1.03 ± 0.25 | 1.01 ± 0.27 | 0.663 |
| LDL-C (mmol/L) | 2.60 ± 0.79 | 2.97 ± 1.17 | 0.002 |
| Homocysteine (umol/L) | 15.19 ± 6.84 | 15.89 ± 7.93 | 0.476 |
| **Angiographic findings** |  |  |  |
| Chronic total occlusions, n (%) | 54 (11.64%) | 14 (23.73%) | 0.009 |
| Number of stent (/patients) | 1 (1, 2) | 2 (1, 2) | 0.013 |
| Length of stents (mm/patients) | 27.03 ± 6.35 | 28.00 ± 5.00 | 0.403 |
| Minimal stent diameter (mm) | 3.05 ± 0.46 | 2.81 ± 0.33 | <0.001 |
| **Medications at discharge** |  |  |  |
| Aspirin, n (%) | 464 (100.00%) | 59 (100.00%) | >0.99 |
| Clopidogrel/Ticagrelor, n (%) | 464 (100.00%) | 59 (100.00%) | >0.99 |
| Statin, n (%) | 460 (99.14%) | 59 (100.00%) | 0.474 |
| β-block, n (%) | 418 (90.09%) | 51 (86.44%) | 0.386 |
| ACEI/ARB, n (%) | 323 (69.61%) | 36 (61.02%) | 0.180 |
| Oral hypoglycemic drugs, n (%) | 105 (22.63%) | 13 (22.03%) | 0.918 |

^#^ Data are shown as mean ± SD, median (IQR), or n (%). Abbreviations: ISR, in-stent restenosis; BMI, body mass index; eGFR, estimated glomerular filtration rate; FPG, fasting plasma glucose; HbA1c, hemoglobin A1c; TC, total cholesterol; HDL-C, high-density lipoprotein-C; LDL-C, low-density lipoprotein-C; SUA, serum uric acid; ACEI/ARB, angiotensin-converting enzyme inhibitor/angiotensin receptor blocker.

Table S2. Multiple-imputation analysis which is based on 5 replications and the Markov-chain Monte Carlo method in the SAS multiple imputation procedure^†^.

| **Data** | **B** | **SE** | **OR (95%CI)** | ***P* value** |
| --- | --- | --- | --- | --- |
| Pre-imputation | 0.526 | 0.249 | 1.69 (1.04, 2.75) | 0.034 |
| Imputation 1 | 0.464 | 0.237 | 1.59 (1.00, 2.53) | 0.050 |
| Imputation 2 | 0.485 | 0.240 | 1.62 (1.01, 2.60) | 0.043 |
| Imputation 3 | 0.501 | 0.243 | 1.65 (1.03, 2.66) | 0.038 |
| Imputation 4 | 0.480 | 0.240 | 1.62 (1.01, 2.58) | 0.044 |
| Imputation 5 | 0.475 | 0.238 | 1.61 (1.01, 2.57) | 0.046 |
| Pooled estimates | 0.481 | 0.240 | 1.62 (1.01, 2.60) | 0.045 |

^†^All models adjusted the same covariates, including age, gender, body mass index, total cholesterol, low-density lipoprotein-C, hypertension, diabetes, length of stents, and minimal stent diameter. Abbreviations: SE, standard error; OR, odds ratio; CI, confidence interval.


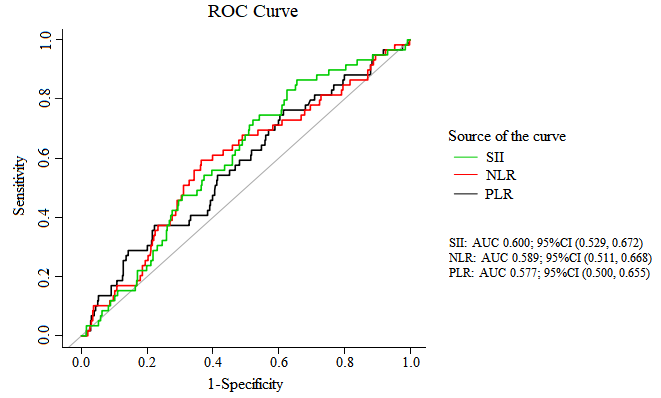


Figure S1. The ROC curve of SII, NLR and PLR for the identification of DES-ISR in patients with ACS. Abbreviations: ROC, receiver operator characteristic; SII, systemic immune inflammation index; NLR, neutrophil/lymphocyte ratio; PLR, platelet to lymphocyte ratio; DES, drug-eluting stent; ISR, in-stent restenosis; ACS, acute coronary syndrome.
